# Shear wave elastography in chronic kidney disease: a pilot experience in native kidneys

**DOI:** 10.1186/s12882-015-0120-7

**Published:** 2015-07-31

**Authors:** Anthony E. Samir, Andrew S. Allegretti, Qingli Zhu, Manish Dhyani, Arash Anvari, Dorothy A. Sullivan, Caitlin A. Trottier, Sarah Dougherty, Winfred W. Williams, Jodie L. Babitt, Julia Wenger, Ravi I. Thadhani, Herbert Y. Lin

**Affiliations:** Department of Radiology, Massachusetts General Hospital, Boston, MA 02114 USA; Department of Medicine, Division of Nephrology, Massachusetts General Hospital, 185 Cambridge St, Suite 8.216, Boston, MA 02114 USA

**Keywords:** Chronic kidney disease, Stiffness, Fibrosis, Shear wave elastography, Ultrasound

## Abstract

**Background:**

There currently is a need for a non-invasive measure of renal fibrosis. We aim to explore whether shear wave elastography (SWE)-derived estimates of tissue stiffness may serve as a non-invasive biomarker that can distinguish normal and abnormal renal parenchymal tissue.

**Methods:**

Participants with CKD (by estimated GFR) and healthy volunteers underwent SWE. Renal elasticity was estimated as Young’s modulus (YM) in kilopascals (kPa). Univariate Wilcoxon rank-sum tests were used.

**Results:**

Twenty-five participants with CKD (median GFR 38 mL/min; quartile 1, quartile 3 28, 42) and 20 healthy controls without CKD underwent SWE performed by a single radiologist. CKD was associated with increased median YM (9.40 [5.55, 22.35] vs. 4.40 [3.68, 5.70] kPa; *p* = 0.002) and higher median intra-subject inter-measurement estimated YM’s variability (4.27 [2.89, 9.90] vs. 1.51 [1.21, 2.05] kPa; *p* < 0.001).

**Conclusions:**

SWE-derived estimates of renal stiffness and intra-subject estimated stiffness variability are higher in patients with CKD than in healthy controls. Renal fibrosis is a plausible explanation for the observed difference in YM. Further studies are required to determine the relationship between YM, estimated renal stiffness, and renal fibrosis severity.

**Electronic supplementary material:**

The online version of this article (doi:10.1186/s12882-015-0120-7) contains supplementary material, which is available to authorized users.

## Background

Chronic kidney disease (CKD) is a major public health challenge [1]. There are currently 19 million adults in the US in early stages of CKD and over 640,000 adults with end stage renal disease (ESRD) [2–4]. Projections suggest that the number of patients who will require dialysis or transplantation for kidney failure will rise to over 2 million people by 2030 [3, 4].

Advanced CKD is associated with increased morbidity and mortality [5]. Therefore, it is important to quantify CKD severity. Currently, CKD is staged based on estimated glomerular filtration rate (eGFR), derived from serum creatinine values in one of several formulas [2]. Limitations of this measure are well documented, including confounding by race, gender, and muscle mass [6].

Intra-renal fibrosis is a final common pathway for all CKD, with fibrosis degree correlated with disease severity [7–9]. Non-focal renal biopsy is the only method in current clinical use for the evaluation of intra-renal fibrosis. However, non-focal renal biopsy has significant disadvantages: (1) it is invasive, with risk of major complications, (2) it is expensive, with costs of greater than $1000 (US) per procedure, and (3) it is subject to sampling error, as the biopsy core/s comprise a small fraction of the renal parenchyma, and highly fibrotic kidneys often have insufficient glomerular tissue on biopsy samples to permit accurate histopathologic diagnosis [10–12].

Shear wave elastography (SWE) is an emerging ultrasound technique that permits the non-invasive measurement of tissue stiffness. SWE uses focused acoustic energy pulses to produce microscopic tissue displacement, which induces perpendicular shear waves that are sonographically tracked as they progress through tissue. Stiffer tissues have been shown to have increased shear wave velocities. Estimates of tissue Young’s modulus (YM), measured in kilopascals [kPa] can be derived from shear wave velocity, where higher values correlate with a higher degree of fibrosis [13, 14]. This technique has been FDA approved for use in liver disease and has high sensitivity and specificity to discriminate between normal and cirrhotic liver [15]. Variations of SWE have been used to study other organs including breast, thyroid, prostate, and renal allografts [16–21]. Prior human and animal studies have shown a correlation between SWE estimates of renal YM and presence of CKD or fibrosis [22, 23].

Shear wave elastography has only been used in two prior studies in native kidneys and has not yet been used to examine a heterogeneous population of CKD in the United States [22, 24]. There are limitations to these prior studies, including lack of non-diseased comparison group [22]. In this pilot study, we aim to explore whether SWE-derived estimates of tissue YM may serve as a non-invasive biomarker that can distinguish normal and abnormal renal parenchymal tissue.

## Methods

### Patient population

For this cross sectional pilot study, subjects were recruited from the outpatient renal clinic panels at an academic tertiary care center from March 2014 to September 2014. Inclusion criteria for subjects with CKD included: age greater than 18 years, eGFR less than 60 mL/min by the IDMS-traceable, 4-variable MDRD equation [25] or known diagnosis of CKD, and consent to undergo renal ultrasound. Exclusion criteria included body mass index (BMI) greater than 35 kg/m^2^, pregnancy or nursing status, or any condition that impeded visualization of the kidney by ultrasound. Healthy control subjects were screened for the absence of common medical conditions including CKD (and/or eGFR < 60 mL/min), hypertension, diabetes, and cardiovascular disease. Control subjects were recruited at the study site. Inclusion criteria for healthy subjects included: age greater than 18, BMI less than 35 kg/m^2^, not pregnant or nursing, and structurally normal kidneys on traditional renal ultrasound. Participants were not included or excluded on the basis of race, gender, or ethnicity. Demographic and medical information was taken from electronic medical record or by interview. Past medical history and etiology of CKD were determined by the participants’ treating providers and were extracted from medical documentation. Lab values were taken from the electronic medical record within one month of undergoing SWE, or if values were unobtainable, a study nurse performed a separate blood draw at the time of SWE. Study data were collected and managed using REDCap electronic data capture tools hosted at the Harvard Clinical and Translational Science Center [26].

### Shear wave elastography

**Fig. 1:**
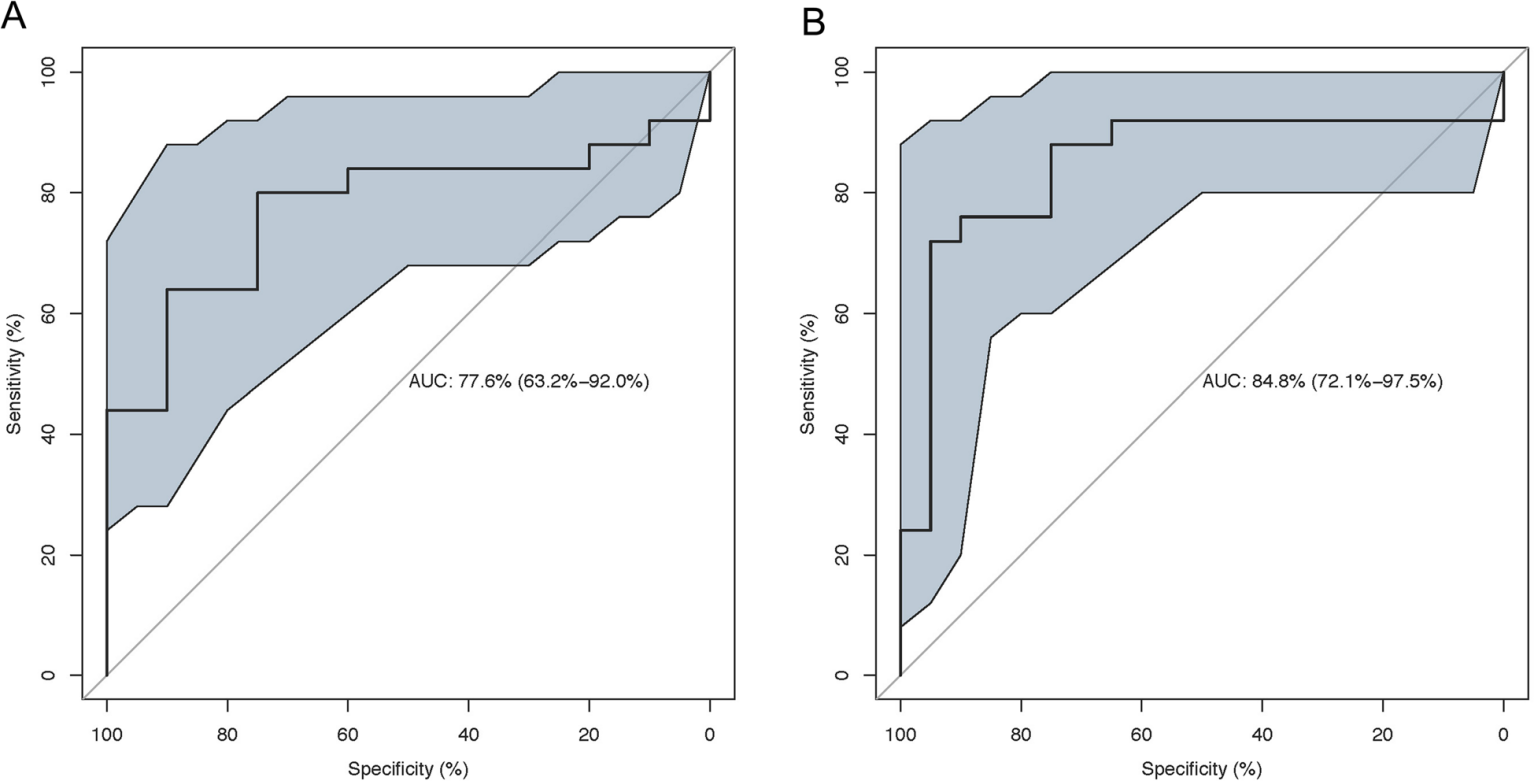
ROC Curves for Detecting Presence of CKD. Panel (**a**): Median Young’s Modulus (*p* = 0.018). Panel (**b**): Intra-subject Variability of Young’s Modulus (*p* = 0.002). P values were derived from logistic regression

Shear wave elastography was performed with a curved 2–5 MHz broadband transducer on a two-port Aixplorer ultrasound system (Supersonic Imagine, Paris, France). A single board-certified radiologist (AES) with 13 years of sonography experience performed all SWE scans for this study. Participants were scanned in the typical manner renal sonographic images are obtained clinically, in the position offering the shortest distance to either kidney, typically the left decubitus or supine positions. Body position was not recorded. SWE measurements were obtained in a single region of interest (minimum diameter 6 mm) an area of renal parenchyma at least 1 cm deep of the capsule in the renal cortex, with specific avoidance of renal pyramids as the operator was able. Measurements where obtained where the acoustic window was optimal, typically in the lower renal pole. Distance from the skin to the region of interest was recorded as kidney depth and listed in Table 1 for cases and controls. Eight to twelve readings were taken per subject and a median SWE value was recorded as YM in kPa (Fig. 1). YM was calculated by Aixplorer software under the assumption of target tissue at body temperature using the formula E = ρ x c^2^, where E is tissue elasticity in kPa, ρ is tissue density in kg/m^3^ and *c* is shear wave velocity in m/s [27]. SWE measurements were obtained at end-expiration. All patients had confirmation of absence of hydronephrosis by traditional ultrasound prior to undergoing SWE. In all cases, imaging began with the right kidney. If the kidney was readily accessible SWE measurements were obtained. If the right kidney was deep or the acoustic window was considered suboptimal by the radiologist, SWE measurements were obtained from the left kidney. Eighty eight percent of participants underwent SWE on the right kidney.

**Table 1:** Demographic information

	CKD (*n* = 25)	Control (*n* = 20)
Age (years)	61 (56, 70)	34 (29, 49)
Male Gender	16 (64 %)	5 (25 %)
Ethnicity^a^		
Non-Hispanic	20 (83 %)	18 (90 %)
Hispanic	4 (17 %)	2 (10 %)
Race		
White	20 (80 %)	15 (75 %)
Other	5 (20 %)	5 (25 %)
Height (cm)	170.2 (163.0, 177.8)	165.0 (162.8, 169.0)
Weight (kg)	76.0 (71.0, 88.0)	65.8 (61.1, 69.0)
BMI (kg/m^2^)	26.1 (24.8, 28.4)	23.4 (22.1, 24.0)
Kidney Length (cm) ^b^	10.35 (9.16, 10.95)	10.47 (10.10, 11.07)
Kidney Depth to Region of Interest (cm)	3.60 (1.64, 5.56)	3.15 (2.45, 3.85)
Hematocrit^c^	38.2 (33.6, 40.2)	40.8 (39.6, 41.1)
BUN (mg/dL) ^d^	30 (18, 38)	13 (11, 15)
Creatinine (mg/dL) ^d^	1.74 (1.42, 2.38)	0.90 (0.79, 0.96)
GFR (mL/min)	38 (28, 42)	>60
CKD Stage		
CKD Stage 1-2	1 (4 %)	
CKD Stage 3	17 (68 %)	
CKD Stage 4	5 (20 %)	
CKD Stage 5	2 (8 %)	
Cause of CKD		
Diabetes/Hypertension	13 (52 %)	
IgA Nephropathy	3 (12 %)	
Renovascular Disease	1 (4 %)	
Other Known Diagnosis	6 (24 %)	
Unknown Diagnosis	2 (8 %)	
Other Medical History		
Hypertension	24 (96 %)	
Hyperlipidemia	18 (72 %)	
Diabetes	7 (28 %)	
Gout	5 (20 %)	
Coronary Artery Disease	5 (20 %)	
Congestive Heart Failure	5 (20 %)	
Vascular Disease	4 (16 %)	
Hypothyroidism	4 (16 %)	
Prostatic Hypertrophy	3 (12 %)	

All continuous variables are given as medians (quartile 1, quartile 3). CKD stage calculated by MDRD equation. ^a^ *N* = 24 for CKD. ^b^ *N* = 23 for CKD and *N* = 18 for controls. ^c^ *N* = 20 for CKD and *N* = 9 for controls. ^d^ *N* = 9 for controls

### Statistical analysis

The characteristics of the study participants by CKD and control group status were presented using median (quartile 1, quartile 3) or numbers (percentages). The median value for estimated tissue YM was selected due to the non-normality of individual measurements. The main outcome (median estimated tissue YM in kPa) was reported as a continuous variable and was compared between exposure variables using Wilcoxon Rank-Sum tests. Intra-subject variability was examined by comparing difference of individual readings from the median YM for each subject.

The diagnostic performance of SWE for distinguishing normal renal parenchyma from renal parenchyma affected by CKD was assessed using a univariate logistic regression model to construct receiver operating characteristic (ROC) curves. ROC analyses were performed to determine a cut point of SWE that would correctly classify the maximum number of participants based on sensitivity and specificity values. Wald asymptotic 95 % confidence limits are presented for sensitivity and specificity values. Percentile method 95 % confidence intervals for ROC curves were generated with 2000 replicate samples using the pROC package in R version 3.0.2 (Vienna, Austria) [28, 29]. Comparisons of area under the curves were performed by use of a contrast matrix to take differences of the area under the empirical ROC curves. Pearson correlation coefficients were used to assess the association between continuous exposure variables. Stratified analysis of the control and CKD groups were performed to evaluate for potential significant confounders of SWE values. SAS version 9.4 (Cary, NC) was used for all other non-ROC-related statistical analysis. Two-tailed p values of less than 0.05 were deemed statistically significant.

### Ethics statement

Written informed consent was obtained from all study participants. All responses and patient/provider information were de-identified except to members of the research team. The Partners Human Research Committee for human subjects approved the study. All clinical investigation was conducted according to the principles expressed in the Declaration of Helsinki.

## Results

Twenty-five subjects with CKD (“cases”) and 20 healthy subjects (“controls”) were enrolled. Cases were mostly male (64 %), white race (80 %), and of non-Hispanic origin (83 %). For cases, median GFR was 38 (quartile 1, quartiles 3: 28, 42) mL/min; median Cr was 1.74 (1.42, 2.38) mg/dL (normal range: 0.6-1.50 mg/dL). The majority of cases had CKD stage III or IV (88 %). A sensitivity analysis excluding the single patient with stage I/II CKD did not affect primary outcomes. The most common cause of CKD was diabetes or hypertension (52 %). Controls were mostly female (75 %), white race (75 %), and of non-Hispanic origin (90 %). Median BMI was 26.1 (24.8, 28.4) kg/m^2^ in cases and 23.4 (22.1, 24.0) kg/m^2^ in controls. Median age was 61 (56, 70) years for cases and 34 (29, 49) years for controls. Median kidney length was 10.35 (9.16, 10.95) cm in cases and 10.47 (10.10, 11.07) cm in controls (Table 1).

**Fig. 2:**
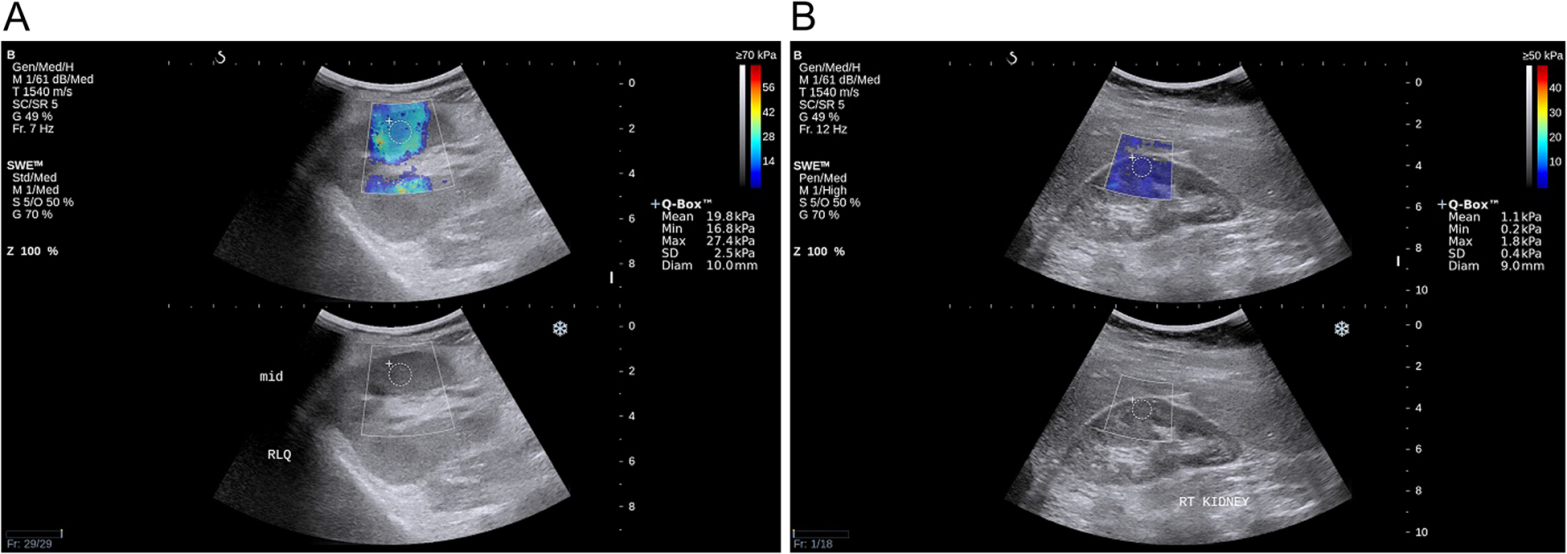
Representative Images of Shear Wave Elastography. Panel (**a**): right kidney of a subject with CKD. Panel (**b**): right kidney of a control subject

**Fig. 3:**
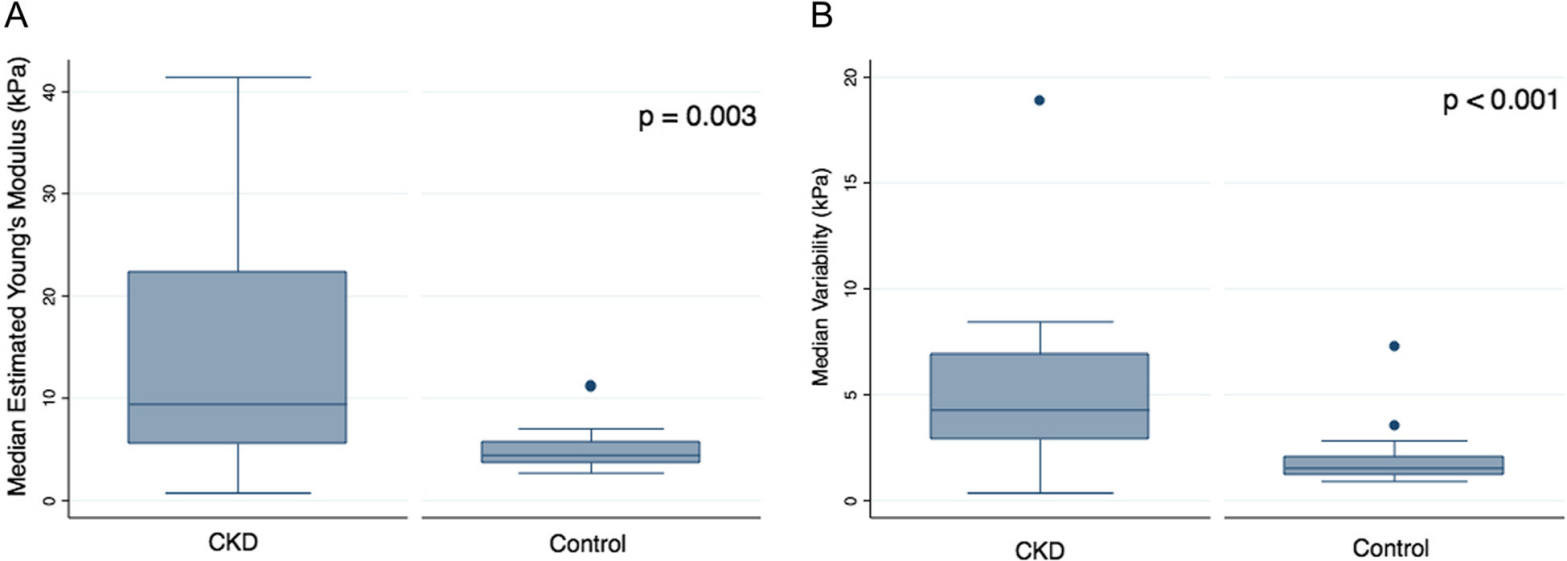
Median Estimated Young’s Modulus in CKD versus Controls (Panel **a**). Median Variability in Intra-subject SWE Readings in CKD and Controls (Panel **b**)

There was a significantly higher median estimated tissue YM for cases compared to controls (*p* = 0.002): median values were 9.40 (5.55, 22.35) kPa and 4.40 (3.68, 5.70) kPa, respectively (Fig. 2). Using a cutoff of 5.3 kPa for median estimated tissue YM, the area under the ROC curve to distinguish CKD from non-CKD state was 0.78 (95 % CI 0.63-0.92; *p* = 0.02) with a sensitivity and specificity of 80 % (95 % CI 64 %-96 %) and 75 % (95 % CI 56 %-94 %), respectively (Fig. 3a). Median intra-subject variability of individual estimated YM (distance from the median YM for each subject) was larger in cases compared to controls (3.88 [2.88, 5.13] vs. 1.41 [1.14, 2.13] kPa; *p* < 0.001). Using a cutoff of 2.8 kPa for intra-subject variability, the area under the ROC curve to distinguish diseased from healthy renal parenchyma was 0.85 (95 % CI 0.72-0.98; *p* = 0.002) with a sensitivity and specificity of 76 % (95 % CI 55 %-91 %) and 90 % (95 % CI 68 %-99 %), respectively (Fig. 3b). The estimated areas under the ROC curve for distinguishing healthy and diseased renal cortex using median estimated tissue YM or intra-subject variability were not significantly different (*p* = 0.15).

Among controls, only race was significantly associated with estimated tissue YM (*p* = 0.01). Among cases, estimated tissue YM was associated with female gender (*p* = 0.03), kidney depth (*p* = 0.02), height (*p* = 0.04), weight (*p* = 0.001), and BMI (*p* = 0.045). There was no correlation between estimated tissue YM and age or kidney length in either group (Table 2). For stratified analysis, potential confounders were dichotomized into high versus low values at their respective medians. Among cases, kidney depth was the only potential confounder that had significantly different values: 5.5 kPa [IQR 12.1] with kidney depth greater than or equal to 3.6 cm versus 12.3 kPa [IQR 18.0] with kidney depth less than 3.6 cm; *p* = 0.02. In the control group, BMI was the only potential confounder that had significantly different values: 5.1 kPa [IQR 2.5] with BMI greater than or equal to 23 kg/m^2^ versus 3.9 kPa [IQR 1.1] with BMI less than 23 kg/m^2^; *p* = 0.01 (Additional fie 1: Table S1).

**Table 2:** Evaluation of Potential Influences on SWE in CKD and Control Groups

Factors	CKD		Control	
	r	P value	r	P value
Age	0.370	0.07	0.325	0.16
Height	−0.423	0.04	−0.235	0.31
Weight	−0.556	0.004	0.033	0.90
BMI	−0.404	0.045	0.251	0.29
Kidney Length	−0.257	0.24	−0.238	0.34
Kidney Depth	−0.525	0.007	−0.336	0.15
Hematocrit^a^	−0.014	0.95	−0.447	0.23
Creatinine	−0.123	0.56		
eGFR	−0.018	0.93		
BUN	−0.084	0.69		
	Young’s modulus		Young’s modulus	
Race		0.58		0.01
White	10.70 (5.08, 24.85)		4.85 (4.30, 6,85)	
Other	7.60 (7.50, 10.50)		3.10 (2.70, 3.90)	
Gender		0.04		0.15
Male	6.60 (4.10, 12.33)		3.55 (3.10, 4.20)	
Female	22.35 (9.40, 25.80)		4.50 (3.90, 6.20)	
Ethnicity		0.87		0.20
Non-Hispanic	10.70 (5.78, 23.13)		4.35 (3.55, 4.90)	
Hispanic	5.58 (4.50, 8.05)		6.03 (5.20, 6.85)	

Correlation coefficient (r) given for continuous variables. Median Young’s modulus (quartile 1, quartile 3) in kPa given for categorical variables. Abbreviations: BMI (body mass index), eGFR (estimated glomerular filtration rate), BUN (blood urea nitrogen). ^a^ *N* = 9 for controls

## Discussion

Our results suggest estimated tissue YM can be used to non-invasively distinguish renal tissue affected by CKD from normal renal tissue, even when kidney size does not differentiate the two conditions. Of the two prior human studies examining SWE in native kidneys, one also reported a correlation between estimated tissue YM and presence of CKD [24]. The second study, of Chinese subjects primarily with early stage CKD, was designed to look for differences between CKD subgroups and did not include healthy patients as a comparator [22]. Except for stage V, neither of these studies detected a correlation between estimated tissue YM and CKD stage. This is unsurprising; the relatively small number of subjects in these studies suggests they were insufficiently powered to do so. The potential of SWE to detect diffuse renal disease is clinically relevant, as conventional B mode sonography is well known to be insensitive for the detection of diffuse renal disease, and is presently used primarily for the exclusion of hydronephrosis [30].

In our study, we make two important assumptions: (1) we assumed CKD would alter tissue stiffness in a way that could be detected by SWE. We considered this biologically plausible, as renal parenchymal fibrosis and inflammation are known to occur in CKD, and fibrosis is known to alter tissue SWE estimates of tissue stiffness in other organs [16–19]. Our study did not include patients who underwent contemporaneous kidney biopsy, so we did not directly measure the relationship between histologic measures of fibrosis and renal stiffness. Since inflammation, fibrosis, and renal perfusion abnormalities all contribute to the clinical CKD syndrome, it is unlikely that histologic measures of renal fibrosis alone would have been an appropriate reference standard.

The majority of prior studies examining variations of SWE technology and renal fibrosis are in renal transplants, and support a positive correlation between SWE estimates of renal stiffness and pathologic fibrosis or eGFR, [19–21, 24, 31, 32] though at least two studies (including one examining native kidneys) did not find a significant correlation [22, 33]. Potential explanations for this include SWE technology variation, type II error owing to small sample sizes, incorrect estimation of fibrosis by biopsy, which is known to be an imperfect reference standard, [34] operator variability, or a true lack of correlation.

A second assumption is that CKD is the cause of tissue stiffness, rather than other potential confounders. Our study identified associations between estimated renal tissue YM and race, gender, kidney depth height, weight, and BMI, albeit not in both case and control groups. Prior studies of renal SWE have identified several potential confounders, including bladder pressure, [23] renal blood flow, [23] BMI, [21] kidney depth, [21] surrounding fluid accumulation, [21] and age [24]. With the exception of BMI and kidney depth, none of these factors appear across multiple studies, suggesting these potential confounding effects may be small or inconsistent. In a large study of healthy subjects undergoing SWE, there was no difference in renal cortex readings between men and women [35]. The effect of potential confounders, such as gender and renal blood flow, remains a potential area of future study for SWE.

When considering these assumptions, it is relevant to note that tissue YM, similar to other physical properties of tissue, such as weight, viscosity, radiodensity, and acoustic impedance, represents bulk tissue properties, and is therefore representative of composite endpoints produced by tissue content, structure, and microenvironment. It is probable that additional situation-specific tissue microenvironment factors, such as inflammation, may confound SWE estimates of tissue YM. This is known to be the case in liver disease, where hepatic inflammation has been shown to increase tissue stiffness estimates [36]. Despite this, the clinical utility of elastography for liver fibrosis staging is now well established, and SWE is used clinically to differentiate early and advanced liver fibrosis without biopsy [15]. We anticipate SWE may similarly have great utility in diffuse renal disease as it has the potential both to reduce biopsy use, and to permit repeated non-invasive direct estimates of renal parenchymal disease severity. Ultimately, such a tool could potentially be used to track renal fibrosis progression and permit therapy individualization in a manner that is presently not possible.

Our study supports prior evidence that the relationship between SWE estimates of tissue YM and renal fibrosis severity may not be as robust as that seen in liver disease. For example, the area under ROC curve for estimates of YM has been reported to be as high as 0.98 in liver disease [15] versus 0.78 in our study and 0.75 in Guo et al. [24]. There are several potential reasons for this, including: (1) greater kidney depth from the skin surface compared with the liver; (2) the more rounded renal shape, which may cause refraction of acoustic displacement pulses with greater variation in renal parenchymal shear wave generation; and (3) larger variability in the reference standards used for the quantitation of renal fibrosis than in the relatively simpler liver fibrosis staging METAVIR classification.

We showed increased variability in individual SWE readings in the CKD group. There are two potential reasons for this: (1) SWE may be less precise in stiffer tissue. A larger standard deviation and range of readings in subgroups with advanced fibrosis were observed in prior liver and kidney studies supporting this notion [15, 19]. (2) Alternatively, there is an intriguing possibility that renal parenchymal fibrosis results in true increased heterogeneity of the tissue YM. If validated, tissue stiffness heterogeneity could prove a valuable biomarker of fibrosis severity, and add additional explanatory power to this new technology. Interestingly, the area under the ROC curve for variability of measurement was better compared to the estimation of YM itself, though this was not statistically significant, and the two predictors together did not create a statistically significant combined model. Other factors, such as probe type, tissue depth, and operator technique are still being explored as explanations of variability in measurements for this new technology [37–39]. Regardless of the reason for measurement variation, it is clear that judicious selection of clinical outcomes and sample size will be necessary in future studies to supply sufficient power. Studies targeted at fibrosis staging dichotomized at clinically relevant cut-offs may be more likely to be productive than attempts to establish a linear relationship between fibrosis stage and tissue stiffness.

One should interpret these results within the context of the limitations of our study: (1) as expected for a pilot study, we were only able to capture a small cross section of the large and heterogeneous CKD population. Half of CKD subjects had diabetic or hypertensive kidney disease, though it is not known if the disease process driving CKD has an independent effect on YM. (2) We were not able to control for all factors described as potential confounders in prior studies. Most notably, we had no measure of renal blood flow, which was out of the scope of this study. Only one subject had known renal vascular disease, and excluding this subject did not affect our conclusions. Kidney depth may have also influenced shear wave readings, which could not be controlled for in this pilot study. (3) Age has been identified as a confounder of YM in prior studies. The median age of the cases was greater than that of the controls in our study, which may have biased the stiffness estimates upward in our case cohort. However, one prior study, [24] advancing age was moderately inversely correlated with renal stiffness. This suggests that the observed higher renal stiffness in the cases was not due to relatively higher age in this group. (4) We also used the known imperfect reference standard of eGFR to estimate CKD severity, and did not have renal biopsy data available to quantify fibrosis histologically. (5) We did not measure inter-observation variance given all SWE studies were performed by a single radiologist. Despite these limitations, we believe our pilot study shows the potential of SWE to expand the role of ultrasound in CKD beyond the exclusion of hydronephrosis to the non-invasive and cost-effective staging of diffuse renal disease.

## Conclusions

SWE-derived estimates of renal stiffness and intra-subject estimated stiffness variability are higher in patients with CKD than in healthy controls. Renal fibrosis is a plausible explanation for the observed difference in YM. Shear wave elastography may be a low-cost way to provide additional diagnostic information in CKD. Further studies are required to determine the relationship between YM, estimated renal stiffness, and renal fibrosis severity.

## Additional file

Additional file 1:
**Table S1.** Stratified analysis of potential confounders in CKD and Control Groups. (DOC 54 kb)

## Abbreviations

CKD: Chronic kidney disease
ESRD: End stage renal disease
eGFR: Estimated glomerular filtration rate
SWE: Shear wave elastography
YM: Young’s modulus
kPa: Kilopascals
BMI: Body mass index
ROC: Receiver operating curves
