# Supplementary material for: Shear wave elastography in chronic kidney disease: a pilot experience in native kidneys
Source: BMC Nephrol. 2015 Jul 31;16:119. doi: 10.1186/s12882-015-0120-7 (PMC4521488; doi:10.1186/s12882-015-0120-7)
Supplement: Additional file 1: — Table S1. Stratified analysis of potential confounders in CKD and Control Groups. (DOC 54 kb) [file 12882_2015_120_MOESM1_ESM.doc]

Additional file 1: Table S1: Stratified analysis of potential confounders in CKD and Control Groups

| CKD | Groups | N | SWE | P value |  | Control | Groups | N | SWE | P value |
| --- | --- | --- | --- | --- | --- | --- | --- | --- | --- | --- |
| Age | ≥62 | 13 | 15.5 (22.1) | 0.18 |  | Age | ≥34 | 9 | 4.3 (1.8) | 0.47 |
|  | <62 | 12 | 7.6 (6.5) |  |  |  | <34 | 11 | 4.5 (2.3) |  |
| Height | ≥170 | 13 | 7.6 (7.2) | 0.24 |  | Height | ≥165 | 14 | 4.4 (1.6) | 0.83 |
|  | <170 | 12 | 13.0 (16.4) |  |  |  | <165 | 6 | 4.4 (2.4) |  |
| Weight | ≥76 | 13 | 7.5 (13.1) | 0.14 |  | Weight | ≥66 | 10 | 4.4 (1.4) | 0.57 |
|  | <76 | 12 | 12.3 (18.1) |  |  |  | <66 | 10 | 4.4 (3.2) |  |
| BMI | ≥26 | 13 | 7.8 (19.3) | 0.06 |  | BMI | ≥23 | 12 | 5.1 (2.5) | 0.01 |
|  | <26 | 12 | 12.3 (19.0) |  |  |  | <23 | 8 | 3.9 (1.1) |  |
| Kidney Length | ≥10.4 | 10 | 11.4 (10.1) | 0.64 |  | Kidney Length | ≥10.5 | 9 | 4.5 (1.1) | 0.97 |
|  | <10.4 | 15 | 10.5 (16.8) |  |  |  | <10.5 | 11 | 4.3 (3.6) |  |
| Kidney Depth | ≥3.6 | 13 | 5.5 (12.1) | 0.02 |  | Kidney Depth | ≥3.15 | 10 | 4.4 (1.8) | 0.57 |
|  | <3.6 | 12 | 12.3 (18.0) |  |  |  | <3.15 | 10 | 4.5 (3.1) |  |
| Hct | ≥38.2 | 10 | 7.8 (22.1) | 0.80 |  | Hct | ≥40.8 | 5 | 3.6 (1.8) | 0.27 |
|  | <38.2 | 15 | 12.0 (16.8) |  |  |  | <40.8 | 4 | 5.5 (1.9) |  |
| BUN | ≥30 | 13 | 7.6 (6.5) | 0.24 |  |  | | | | |
|  | <30 | 12 | 14.1 (18.3) |  |  |  |  |  |  |  |
| Creatinine | ≥1.7 | 14 | 6.8 (6.5) | 0.15 |  |  |  |  |  |  |
|  | <1.7 | 11 | 15.5 (18.3) |  |  |  |  |  |  |  |
| eGFR | ≥38 | 14 | 9.1 (16.8) | 0.81 |  |  |  |  |  |  |
|  | <38 | 11 | 9.4 (23.3) |  |  |  |  |  |  |  |

All factors dichotomized at their medians. SWE given as Young’s modulus median (interquartile range). Abbreviations: BMI (body mass index), eGFR (estimated glomerular filtration rate), Hct (hematocrit)
